# Effect of NGR1 on the Atopic Dermatitis Model and its Mechanisms

**DOI:** 10.1515/med-2019-0099

**Published:** 2019-11-10

**Authors:** Mingmei Wang, Jianli Ma

**Affiliations:** 1 Department of Pharmacy, Fourth Medical Center of PLA General Hospita, 51#Fucheng Road, Beijing 100037, China

**Keywords:** Atopic dermatitis, Notoginsenoside R1, inflammation, NF-κB pathway, NLRP3 inflammasome

## Abstract

Atopic dermatitis (AD) is a highly pruritic chronic inflammatory skin disease. Notoginsenoside R1 (NGR1), a unique ingredient of P. notoginseng which is a well-known medicinal herb for its long history of use in traditional Chinese medicine, has been identified to have various biologically active properties that include anti-inflammatory effects. However, the effects of NGR1 on AD remain unclear. Therefore, this study aimed to investigate the effect and mechanism of NGR1 on the in vitro cell model of AD induced by LPS stimulation. RAW264.7 cells were stimulated with 1 μg/ml LPS to establish the in vitro cell inflammation model of AD. RAW264.7 cells were treated with various concentrations of NGR1 (0.1, 1, and 10 μM); then, an MTT assay was performed to determine the cell viability. An ELISA assay detected the levels of pro-inflammatory cytokines (interleukin-1β, IL-1β; interleukin-6, IL-6; tumor necrosis factor-α, TNF-α). Additionally, NO production was measured using a nitrate/nitrite assay kit. Results indicated that LPS induced increases in the levels of TNFα, IL-1β, IL-6, and NO production was significantly reduced by NGR1 treatment in a dose-dependent manner. Further, NGR1 treatment inhibited the activation of the NF-κB pathway, and the NLRP3 inflammasome in LPS stimulated RAW264.7 macrophages. The study data indicated that NGR1 might relieve atopic dermatitis via inhibiting inflammation through suppressing the NF-κB signaling pathway and NLRP3 inflammasome activation.

## 1 Introduction

As a highly pruritic chronic inflammatory skin disease, atopic dermatitis (AD) affects 10%–20% of children globally [1,2]. Characterized by infiltration of inflammatory cells (such as mast cells, eosinophils, and macrophages) into damaged skin [1, 2, 3], the pathogenesis of AD has not been fully elucidated but is thought to result from a complex interaction of immune, genetic, and environmental factors [4]. As a major public health problem, in addition to the pediatric prevalence rate, AD has an adult prevalence rate of 1%–3% [5,6]. Currently, although great progress has been made in the treatment of AD, no highly effective treatment for AD has been developed. Therefore, finding new and effective methods for treating AD is a priority.

Notoginsenoside R1 (NGR1) is a novel didodecadiene 20(S)-protopanaxatriol saponin that has been identified as a unique saponin in Panax notoginseng [7]. A large body of evidence indicates that NGR1 has neuroprotective, cardioprotective, as well as anti-tumor, antioxidant, and anti-inflammatory effects [8, 9, 10, 11, 12, 13, 14]. Current studies have indicated that NGR1 plays a protective role in the treatment of cardiac dysfunction [15,16], acute liver failure [17], diabetic nephropathy [18], colorectal cancer [12], cardiac hypertrophy [19] etc. However, to the best of our knowledge, the effects of NGR1 on AD remain unclear; therefore, we performed the current study.

It is well known that macrophages play a key role in initiating and expanding inflammatory responses [20]. Lipopolysaccharide (LPS) is a major component of the outer membrane of Gram-negative bacteria and is one of the most effective inflammatory initiators that can activate monocytes and macrophages to produce proinflammatory cytokines [21]. In recent years, LPS-induced cellular inflammation models have widely been used in *in vitro* studies of AD [22, 23, 24].

In the present study, we investigated the effect of NGR1 on the *in vitro* cell inflammation model of AD induced by LPS stimulation and explored the underlying mechanism.

## 2 Materials and methods

### 2.1 Cell culture

The RAW264.7 macrophage cell line was purchased from the Chinese Academy of Sciences Cell Bank (Shanghai, China). RAW264.7 cells were grown in DMEM containing 10% fetal bovine serum (FBS; HyClone, Utah, USA) and 1% penicillin-streptomycin. The cells were incubated at 37°C with 5% CO_2_.

### 2.2 AD cell model establishment

To establish the AD cell inflammation model *in vitro*, RAW264.7 cells were stimulated with 1 μg/ml LPS for 24 h. The RAW264.7 cells were incubated in DMEM medium supplemented with 10% FBS for 24 h. Then, in addition to an untreated control sample, three samples of the cells were pretreated with 0.1, 1 or 10 μM notoginsenoside R1 (NGR1); for 2 h in serum-free media; subsequently, the cells were subjected to 1 μg/ml LPS at 37°C for 24 h. Cells were divided into four groups: the control group (RAW264.7 untreated cells); the LPS group (RAW264.7 cells were subjected to 1 μg/ml LPS at 37°C for 24 h); the 0.1 μM group (RAW264.7 cells were pretreated with 0.1 μM NGR1 for 2 h, then subjected to 1 μg/ml LPS at 37°C for 24 h); the 1 μM group (RAW264.7 cells were pretreated with 1 μM NGR1 for 2 h, then subjected to 1 μg/ml LPS at 37°C for 24 h); the 10 μM group (RAW264.7 cells were pretreated with 10 μM NGR1 for 2 h, then subjected to 1 μg/ml LPS at 37°C for 24 h).

### 2.3 Cell viability assay

We performed an MTT assay to determine the cell viability in the present study. In brief, RAW264.7 cells were plated into a 96-well plate at the density of 3×10^5^ cells per well and then treated with or without various concentrations of NGR1 (0.1, 1, and 10 μM) for 24 h. Subsequently, we added 5 mg/ml MTT solution to each well, and the cells were incubated at 37°C for another 4 h. At the end of the experiment, the optical densities (OD) at 590 nm was detected using a microplate reader (BioRad Laboratories, Inc., Hercules, CA, USA). The experiments were repeated 3 times. Cells in this experiment was divided into four groups: the control group (RAW264.7 cells without any treatment); 0.1 μM group (RAW264.7 cells treated with 0.1 μM NGR1 at 37°C for 24 h); 1 μM group (RAW264.7 cells treated with 1 μM NGR1 at 37°C for 24 h); 10 μM group (RAW264.7 cells treated with 10 μM NGR1 at 37°C for 24 h).

### 2.4 NO level detection

RAW264.7 cells were pretreated with concentrations of NGR1 (0.1, 1, and 10 μM) for 2 h in serum-free media; other than the control group, the pretreated cells were then treated with 1 μg/ml LPS at 37°C for 24 h. The level of NO in the supernatants of the cells was determined using the nitrate/nitrite assay kit (Abnova, USA) following the manufacturer’s protocol. Experiments were repeated 3 times.

### 2.5 ELISA assay

To detect the levels of interleukin-1β (IL-1β), interleukin-6 (IL-6) and tumor necrosis factor-α (TNF-α) in the cell culture medium of RAW264.7 cells, ELISA assay was performed. The levels of IL-1β, IL-6 and TNF-α in the cell culture medium of different groups were detected using ELISA kits according to the manufacturer’s instructions. Cells in the control group did not receive any treatment. Each test was performed 3 times.

### 2.6 Reverse transcription quantitative polymerase chain reaction (RT-qPCR)

Total RNA from RAW264.7 cells was extracted using TRIzol (Invitrogen; Thermo Fisher Scientific Inc.) and reverse-transcribed into cDNA using a TaqMan microRNA Reverse Transcription kit (Invitrogen) according to the manufacturer’s protocol. The temperature protocol for the reverse transcription reaction was as follows: 25˚C for 5 min, 42˚C for 60 min, and 80˚C for 2 min. We then used the TaqMan^®^ Universal PCR Master Mix kit (Thermo Fisher Scientific Inc.) to analyze the mRNA levels. Amplification conditions for PCR were as follows: 95˚C for 10 min, 35 cycles of 95˚C for 15 sec, then 55˚C for 40 sec. GAPDH was used as the internal control. Relative gene expression was quantified by the 2^-ΔΔCq^ method [25].

### 2.7 Western blot assay

Protein levels were calculated using western blotting in the present study. Total proteins from cells were extracted using lysis buffer (Beijing Solarbio Science & Technology Co., Ltd., Beijing, China) according to the manufacturer’s protocol. BCA assay (Thermo Fisher Scientific, Inc.) was performed to detect the protein concentrations. Equal amount of proteins (25 μg per lane) were separated by 12% SDS-PAGE, electro-transferred onto PVDF membranes (Millipore, Bedford, MA, USA), blocked with 5% non-fat milk for 1 h at room temperature, and then incubated with the primary antibodies [p-NF-κB (p-p65), NLRP3, ASC, cleaved caspase-1, caspase-1, iNOS, COX2 and β-actin] overnight at 4˚C. After washing 3 times with TBST, the membranes were incubated with a secondary antibody at room temperature for 1 h. Protein bands were visualized using an enhanced chemiluminescence system (Pierce) per manufacturer’s instructions and quantified using Quantity One Version 4.6 Image software (Bio-Rad).

### 2.8 Statistical analysis

Data were displayed as the mean ± standard deviation (SD) from 3 independent experiments. Statistical analyses were performed using SPSS version 19.0 (SPSS Inc., Chicago, USA). Comparisons between groups were performed by Student’s *t*-test or one-way ANOVA followed by NSK test; p<0.05 indicated statistically significance.

## 3 Results

### 3.1 NGR1 had no significant effect on the viability of RAW264.7 macrophages

**Figure 1:**
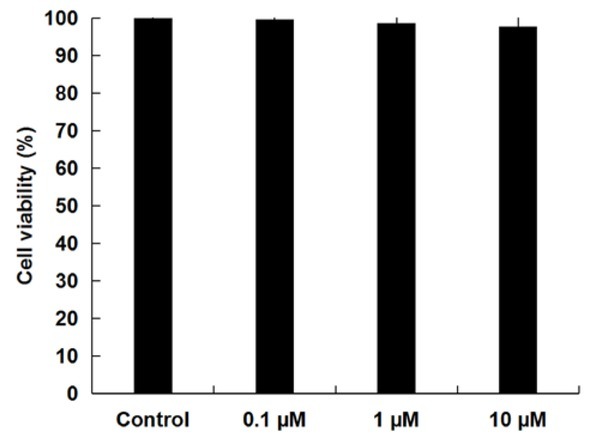
Effect of NGR1 on the viability of RAW264.7 macrophages. RAW264.7 cells were treated with concentrations of NGR1 (0.1, 1, and 10 μM) for 24 h, the cell viability was then determined by MTT assay. Data were displayed as the mean ± SD.

After treatment with various concentrations of NGR1 (0.1, 1, and 10 μM) for 24 h, the cytotoxic effects of NGR1 on RAW264.7 cells were determined by MTT assay. We found that the cell viability of RAW264.7 cells showed no significant difference between groups, indicating that NGR1 had no cytotoxic effect on RAW264.7 cells (Figure 1).

### 3.2 NGR1 reduced LPS-induced pro-inflammatory cytokine secretion in RAW264.7 macrophages

**Figure 2:**
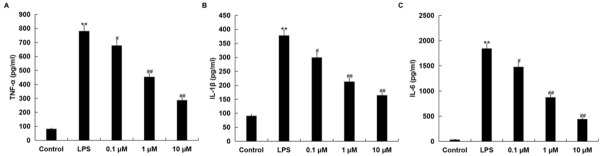
Effects of NGR1 on pro-inflammatory cytokine production in LPS-treated RAW264.7 cells. In addition to an untreated control sample, RAW264.7 cells were pretreated with concentrations of NGR1 (0.1, 1, and 10 **μ**M) for 2 h and then treated with 1 **μ**g/ml LPS at 37˚C for 24 h. The levels of TNF-α, IL-1β, and IL-6 in the culture supernatants of RAW264.7 macrophages were then detected by ELISA. Data were presented as the mean ± SD. **p<0.01 vs. control group; #, ## p<0.05, 0.01 vs. LPS treatment alone group.

We then investigated whether NGR1 had anti-inflammatory effects on AD *in vitro* cell inflammation model; the levels of TNF-α, IL-1β and IL-6 in the culture supernatants of RAW264.7 macrophages were detected using ELISA assay. Results showed that LPS-induced increases of TNF-α, IL-1β and IL-6 were decreased by NGR1 treatment in a dose-dependent manner (Figure 2).

### 3.3 NGR1 reduced LPS-induced NO production in RAW264.7 macrophages

**Figure 3:**
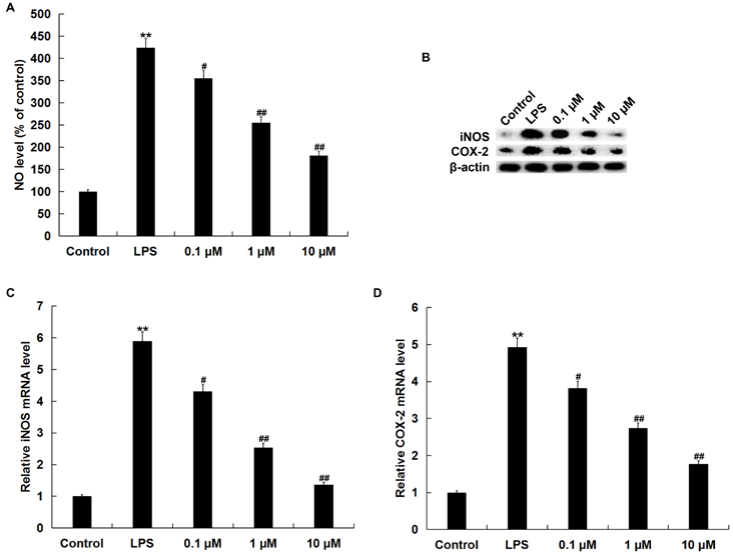
Effects of NGR1 on NO production, iNOS and COX2 expression in LPS-treated RAW264.7 cells. In addition to an untreated control sample, RAW264.7 cells were pretreated with various concentrations of NGR1 (0.1, 1 and 10 **μ**M) for 2 h and then treated with 1 **μ**g/ml LPS at 37˚C for 24 h. A: the secretion of NO was detected using the nitrate/nitrite assay kit; B: protein levels of iNOS and COX2 were measured by western blot assay; C and D: mRNA levels of iNOS and COX2 were measured by qRT-PCR. Data were expressed as the mean ± SD. **p<0.01 vs. control group; #, ## p<0.05, 0.01 vs. LPS treatment alone group.

Compared with the control group, the level of NO increased significantly in the LPS-treated RAW264.7 macrophages; this increase was reversed by NGR1 administration in a dose-dependent manner (Figure 3A).

Because iNOS and COX2 are closely related to NO production, we examined the protein and mRNA levels of iNOS and COX2. Figure 3B-D shows that the protein and mRNA levels of iNOS and COX2 were significantly enhanced in RAW264.7 macrophages by LPS treatment, whereas NGR1 markedly reduced the protein and mRNA levels of iNOS and COX2 (Figure 3B-D).

### 3.4 NGR1 inhibited the activation of NF-κB pathway in LPS-stimulated RAW264.7 macrophages

**Figure 4:**
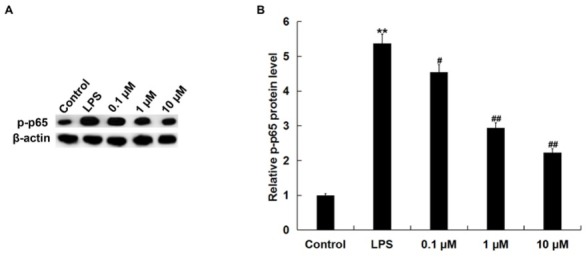
Effects of NGR1 on phosphorylation of NF-κB/p65 protein expression in LPS-treated RAW264.7 cells. In addition to an untreated control sample, RAW264.7 cells were pretreated with various concentrations of NGR1 (0.1, 1, and 10 **μ**M) for 2 h and then treated with 1 **μ**g/ml LPS at 37˚C for 24 h. A: the protein level of p-p65 was detected using western blotting; B: the relative protein level of p-p65 was analyzed and presented as the fold of the control. Data were expressed as the mean ± SD. **p<0.01 vs. control group; #, ## p<0.05, 0.01, vs. LPS treatment alone group.

Next, we investigated whether NF-κB pathway was involved in the anti-inflammatory effect of NGR1 on LPS induced in RAW264.7 macrophages. As shown in Figure 4A and B, LPS stimulation significantly increased the protein level of p-p65 in RAW264.7 cells. However, NGR1 pretreatment significantly attenuated the enhancement of p-p65.

### 3.5 NGR1 inhibited the activation of NLRP3 inflammasome in LPS-stimulated RAW264.7 macrophages

**Figure 5:**
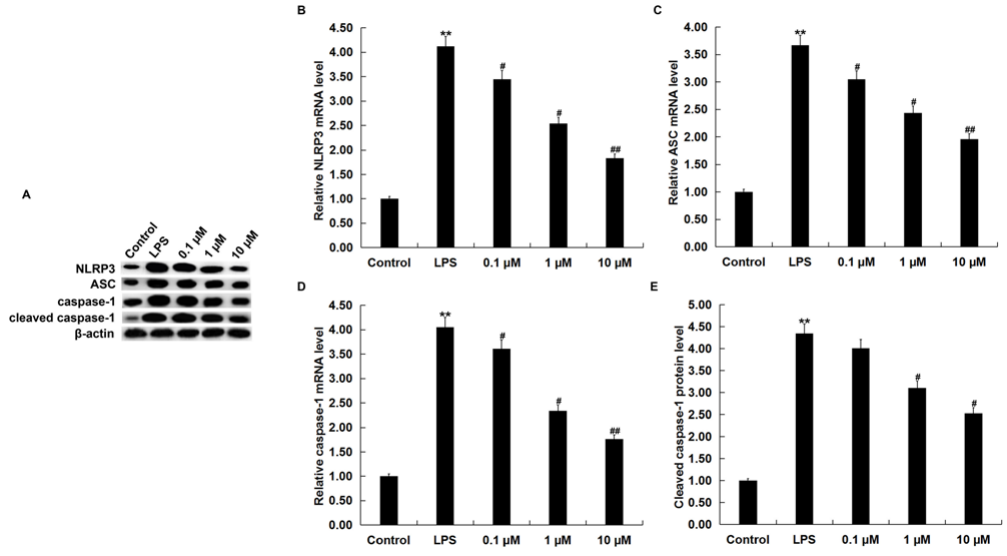
Effects of NGR1 on NLRP3 inflammasome in LPS-treated RAW264.7 cells. In addition to an untreated control sample, RAW264.7 cells were pretreated with various concentrations of NGR1 (0.1, 1, and 10 **μ**M) for 2 h and then treated with 1 **μ**g/ml LPS at 37˚C for 24 h. A: the protein levels of NLRP3, ASC, cleaved caspase-1, and caspase-1 were detected using western blotting; B, C, D: the mRNA levels of NLRP3, ASC, and caspase-1 were detected using qRT-PCR; E: the relative protein level of cleaved caspase-1 was analyzed and presented as fold of control. Data were expressed as the mean ± SD. **p<0.01 vs. control group; #, ## p<0.05, 0.01 vs. LPS treatment alone group.

Finally, we investigated the effect of NGR1 on NLRP3 inflammasome activation in LPS induced RAW264.7 cells. Compared with the control group, the protein levels of NLRP3, ASC, cleaved caspase-1, and caspase-1 were all markedly enhanced in the LPS-treated RAW264.7 cells. NGR1 treatment notably inhibited LPS induced enhancement of NLRP3, ASC, cleaved caspase-1, and caspase-1 protein levels (Figure 5A and E). Additionally, the LPS-enhanced NLRP3, ASC, and caspase3 mRNA levels were significantly reduced by NGR1 treatment (Figure 5B-D).

## 4 Discussion

The present study demonstrated that NGR1 significantly reduced the levels of pro-inflammatory cytokines and NO production that were enhanced by LPS stimulation in RAW264.7 cells. Additionally, the activation of the NF-κB pathway and NLRP3 inflammasome in RAW264.7 cells caused by LPS stimulation was repressed by NGR1 treatment. Therefore, the present study identified a potential new method for the treatment of AD.

Currently, AD seriously affects the quality of life of patients [26]. In recent years, although some drugs (antihistamines, steroids, and immunosuppressants) are available for the treatment of AD, their long-term efficacy is limited by side effects. In this study, we investigated the effect of NGR1, which has been identified to have anti-inflammatory effects [27], on AD *in vitro* cell inflammation model.

We firstly determined whether NGR1 had a cytotoxic effect on RAW264.7 cells, and RAW264.7 cells treated with various concentrations of NGR1 (0.1, 1 and 10 μM) for 24 h. The results indicated that NGR1 had no cytotoxic effect on RAW264.7 cells. Then, to investigate the effect of NGR1 on AD, an *in vitro* cell inflammation model of AD was established by stimulating RAW264.7 cells with LPS for 24 h. Consistent with the previous study [23], our data showed that LPS stimulation significantly enhanced the production of pro-inflammatory cytokines (TNF-α, IL-1β and IL-6) and NO.

iNOS, a major producer of NO, plays an important role in the progression of inflammatory diseases, including AD [28]. COX-2 expression is enhanced in inflammation-related cells after cytokine stimulation during the immune reaction, resulting in the secretion of PGD2 and PGE2, which are involved in the inflammatory response [28]. Also, studies have indicated that LPS treatment could enhance iNOS and COX-2 expression in RAW264.7 cells [29,30]. Therefore, we explored whether the expression of iNOS and COX2 could be affected by NGR1. Consistent with previous studies [29,30], our results indicated that LPS treatment significantly increased the expression of iNOS and COX2, and that these increases were reduced by NGR1 treatment.

NF-κB is a ubiquitously expressed transcription factor that can regulate the expression of many inflammatory cytokines. NF-κB pathway activation in LPS-induced RAW264.7 cells has also been reported [29,30]. In addition, LPS treatment could enhance NLRP3 inflammasome activation in RAW264.7 cells [31]. Studies have also revealed that the NF-κB pathway activation participates in NLRP3 inflammasome activation [32]. Finally, we explored the underlying mechanism of the effect of NGR1 on LPS-treated AW264.7 cells, the NF-κB pathway, and the NLRP3 inflammasome. Consistent with previous studies [29, 30, 31], our findings suggested that LPS treatment significantly activated the NF-κB and NLRP3 inflammasome pathways, whereas NGR1 treatment markedly repressed the activation of the NF-κB pathway and NLRP3 inflammasome.

In summary, the present study demonstrated that NGR1 treatment inhibited the production of pro-inflammatory cytokines and NO in LPS treated AW264.7 cells, which might be mediated by inhibiting the activation of NF-κB/NLRP3 inflammation signaling pathway and might therefore relieve atopic dermatitis. Therefore, NGR1 may be a potential therapeutic agent for treatment of atopic dermatitis.
